# Protective efficacy of P7C3-S243 in the 6-hydroxydopamine model of Parkinson's disease

**DOI:** 10.1038/npjparkd.2015.10

**Published:** 2015-05-21

**Authors:** Héctor De Jesús-Cortés, Adam D Miller, Jeremiah K Britt, Anthony J DeMarco, Mayralis De Jesús-Cortés, Emily Stuebing, Jacinth Naidoo, Edwin Vázquez-Rosa, Lorraine Morlock, Noelle S Williams, Joseph M Ready, Nandakumar S Narayanan, Andrew A Pieper

**Affiliations:** 1 Graduate program of Neuroscience, University of Texas Southwestern Medical Center, Dallas, TX, USA; 2 Department of Psychiatry, University of Iowa Carver College of Medicine, Iowa City, IA, USA; 3 Department of Neurology, University of Iowa Carver College of Medicine, Iowa City, IA, USA; 4 Department of Biochemistry, University of Texas Southwestern Medical Center, Dallas, TX, USA; 5 Department of Free Radical and Radiation Biology, University of Iowa Carver College of Medicine, Iowa City, IA, USA; 6 The Iowa City Department of Veterans Affairs, Iowa City, IA, USA

## Abstract

**Background:**

There are currently no therapeutic options for patients with Parkinson's disease that prevent or slow the death of dopaminergic neurons. We have recently identified the novel P7C3 class of neuroprotective molecules that blocks neuron cell death.

**AIMS:**

The aim of this study was to determine whether treatment with highly active members of the P7C3 series blocks dopaminergic neuron cell death and associated behavioral and neurochemical deficits in the rat 6-hydroxydopamine (6-OHDA) model of Parkinson's disease.

**Methods:**

After unilateral injection of 6-OHDA into the median forebrain bundle, rats were assessed for behavioral function in the open field, cylinder test, and amphetamine-induced circling test. Thereafter, their brains were subjected to neurochemical and immunohistochemical analysis of dopaminergic neuron survival. Analysis was conducted as a function of treatment with P7C3 compounds, with administration initiated either before or after 6-OHDA exposure.

**Results:**

Animals administered P7C3-A20 or P7C3-S243, two of the most advanced agents in the P7C3 series of neuroprotective compounds, both before and after 6-OHDA exposure showed evidence of protective efficacy in all measures. When P7C3-S243 administration was initiated after 6-OHDA exposure, rats also showed protective efficacy in all measures, which included blocking dopaminergic neuron cell death in ipsilateral substantia nigra pars compacta, preservation of dopamine and its metabolites in ipsilateral striatum, and preservation of normal motor behavior.

**Conclusions:**

The P7C3 series of compounds may form the basis for developing new therapeutic agents for slowing or preventing progression of Parkinson's disease.

## Introduction

Parkinson’s disease (PD) is a progressive and currently incurable neurodegenerative disease characterized by death of midbrain dopaminergic neurons in the substantia nigra pars compacta (SNc). The cardinal motor symptoms of PD consist of resting tremor, rigidity, bradykinesia, hypokinesia, akinesia, postural imbalance, and cognitive disturbance, and disease symptoms manifest after about 60–80% of striatal dopamine content is lost, corresponding to a 50–60% loss of SNc dopaminergic neurons.^1^ Unfortunately, there are no therapies that slow or arrest progression of PD. Current treatment is limited to management of early motor symptoms with drugs that enhance dopaminergic signaling, such as l-3-4-dihydroxyphenylalanine or dopamine receptor agonists. With disease progression, however, these symptomatic medications lose efficacy. There is thus a significant unmet need for new medications capable of slowing or preventing PD progression by blocking SNc neuron death.

We have previously reported the discovery of the P7C3 class of neuroprotective agents,^2–4^ which augment synthesis of nicotinamide adenine dinucleotide through activation of the metabolic enzyme nicotinamide phosphoribosyltransferase.^5^ Nicotinamide adenine dinucleotide is an enzyme co-factor that has a central role in metabolism, mitochondrial integrity, and neuronal survival, and lead agents in the P7C3 series have shown potent protective efficacy in preclinical models of aging-associated cognitive decline,^2^ retinal degeneration,^6^ amyotrophic lateral sclerosis,^7^ peripheral nerve degeneration,^8^ elevated hippocampal cell death after stress,^9^ and traumatic brain injury.^10–12^ Importantly, the P7C3 class of molecules is currently being targeted for development of a new class of neuroprotective drugs.^13^ Both the previous lead agent (P7C3-A20) and the recently reported more active drug-like compound P7C3-S243 exhibit potent neuroprotective efficacy in the mouse 1-methyl-4-phenyl-1,2,3,6-tetraydropyridine (MPTP) model of PD.^14,15^ Although MPTP is a valuable model of PD in mice and nonhuman primates, it is limited by virtue of causing a bilateral Parkinson syndrome due to systemic injection, thereby rendering it impossible to conduct side-biased behavioral tests that incorporate uninjured control tissue in the same organism. Therefore, we sought to evaluate efficacy of P7C3-S243 in an additional toxin model of PD: the unilateral 6-hydroxydopamine (6-OHDA) rat model, in which the toxin is directly injected into one side of the nigrostriatal pathway. In this procedure, 6-OHDA selectively destroys catecholaminergic neurons, and has the unique advantage of side-biased motor impairment that corresponds with loss of dopaminergic neurons, such as amphetamine-driven circling and spontaneous motor activity.^16^

## Materials and methods

### Animals

Long Evans rats weighing 250–300 g were used in this study. All animal procedures were performed in accordance with the protocol approved by the University of Iowa Institutional Animal Care and Use Committee. Animals were housed individually following surgery with food *ad libitum*, and had a 12-h light-on–light-off schedule.

### Surgical Procedure

The nigrostriatal dopaminergic pathway that is implicated in PD consists of dopaminergic neurons located in the SNc that extend axons along the median forebrain bundle (MFB) and terminate in the dorsal striatum. To model PD, 6-OHDA is most commonly injected unilaterally into the substantia nigra, MFB, or striatum. We chose to inject the toxin into the MFB because this approach induces degeneration in striatal terminals before dopaminergic neuron cell death occurs,^17^ and we have previously shown that P7C3 compounds block axonal degeneration preceding neuron cell death in traumatic brain injury.^11^ Rats were anesthetized using ketamine/xylazine anesthesia (ketamine: 100 mg/kg, xylazine: 10 mg/kg) and placed into a stereotactic frame secured by ear and nose bars adapted specifically for the rat. To achieve lesioning of the nigrostriatal pathway, 5 μl of 6-OHDA (1 μg/μl in 0.01% ascorbic acid dissolved in saline preventing heat and light exposure) was stereotactically injected into the right MFB (anteroposterior: −2.2, mediolateral: −1.5, dorsoventral: −8.0) using a 5-μl Hamilton Syringe (Sigma, St Louis, MO, USA) with 28-gauge needle. Syringes were lowered into the brain at a rate of 2 mm/min. 6-OHDA was injected at a rate of 1 μl/min, and the syringe was left in place for 5 min after injection before being drawn back at a rate of 2 mm/min. Rats were monitored for 2 h post surgery before returning to the animal housing facility. Two separate groups of control rats received saline sham injections following the same procedures.

### Treatment with P7C3 compounds

P7C3-A20 and P7C3-S243 were prepared as previously described.^2,15^ Rats received either daily 3 days of pretreatment or no pretreatment (injection right after stereotaxic surgery) doses of P7C3-A20 or P7C3-S243 (or vehicle for control). All P7C3 compounds were administered at 10 mg/kg/day via intraperitoneal injection, during brief sedation (15 s) with isoflurane. A growing body of literature has suggested a possible role for isoflurane anesthesia in disrupting the normal blood–brain barrier (BBB).^18–20^ To ascertain whether the isoflurane anesthesia, used during the stereotactic surgical procedure and present when the first injection of P7C3-S243 was administered, could influence the amount of compound that reached the brain, brain levels of P7C3-S243 were determined in the presence and absence of isoflurane after a 10-mg/kg injection intraperitoneally. There was not a statistically significant increase in levels of P7C3-S243 in the brain in the presence of isoflurane. In fact, levels were decreased (area under the curve=141,243 min×ng/g) slightly relative to those observed in the absence of isoflurane (area under the curve=229,915 min×ng/g); however, as plasma levels were also slightly reduced in the presence of isoflurane, the net effect on the blood:brain ratio was negligible (blood:brain=0.45 absence of isoflurane; BBB=0.52 presence of isoflurane). Daily doses were continued 7 days after surgery (including the day of surgery) before beginning behavioral analysis. Test compounds were no longer administered after behavioral analysis was initiated. For cellular studies, both P7C3-A20 and P7C3-S243 were dissolved in 100% dimethyl sulfoxide and cells were incubated with a final 0.1% dimethyl sulfoxide.

### Pharmacokinetic analysis

Detection of P7C3-S243 in plasma and brains of rats injected intraperitoneally with 10 mg/kg compound in the presence and absence of isoflurane was as described previously^15^, with the additional use of Phenomenex (Torrance, CA, USA) Phree columns to further remove phospholipids from plasma and brain homogenates, as described previously.^12^

### Open field

Eight days following surgery, rats were placed in a clean open field container (45 ×45 cm) surrounded by plexiglass walls in a quiet, well-lit room. Motor and exploratory activity was video recorded and tracked for 20 min using Omniplex software (Plexon, Dallas, TX, USA). Plots tracking the rat’s movement were then constructed by importing coordinates generated by Omniplex software into a Microsoft Excel spreadsheet. The container was sanitized with 70% ethanol between each trial and allowed to air dry.

### Cylinder test

Eight days following surgery, rats were placed into a clean, open-top plexiglass cylinder (diameter: 15 cm, height: 19 mm) in a quiet, well-lit room. Motor and exploratory activity was video recorded for 5 min/session. For scoring, three investigators blind to treatment regimen were trained to count the number of rearings. Rearings were scored as an extension of the hindlimbs beyond the typical grooming position while placing paws on the walls of the cylinder in an exploratory manner, as previously described.^21^

### Amphetamine-induced circling

At 12–14 days following surgery, rats received an intraperitoneal injection of (+)-methamphetamine hydrochloride (M8750 Sigma) (3 mg/kg), and were then placed in a clean open field container (45×45 cm) surrounded by plexiglass walls in a quiet, well-lit room. At 30 min after injection, their activity was recorded for a 10-min period. For scoring, three investigators blind to treatment regimen counted the number of 360-degree ipsiversive rotations, as previously described.^22^ The container was sanitized with 70% ethanol between each trial and allowed to air dry.

### Immunofluorescence and tyrosine hydroxylase quantification

Two weeks following surgery, rats were transcardially perfused with sterile saline until the liver could be observed to have been cleared of blood. The forebrain was then carefully extracted and the frontal lobe was dissected for striatal biochemistry (see striatal biochemistry). Mid- and hindbrain tissue were placed in 4% paraformaldehyde for 24 h at 4 °C. Brains were then placed in 30% sucrose at 4 °C for cryoprotection until the tissue had sunk. Thirty-five-μm-thick sections were collected using a sliding microtome. Sections were blocked (10% normal goat serum, 0.3% Triton-X, in 1x PBS) for 1 h and stained for tyrosine hydroxylase (anti-TH; 1:1,000; AB152 Millipore (Darmstadt, Germany) and Alexa Flour 568 Goat anti-rabbit; 1:400; Jackson ImmunoResearch, West Grove, PA, USA) diluted in blocking buffer. Double labeling was visualized using a Leica DM 6000 CFS confocal scope or a Leica DMI 6000B wide-field scope running Leica’s Advanced Application Suite Advanced Fluorescence software (Leica Microsystems, Buffalo Grove, IL, USA). The number of TH-positive cells in the SNc was quantified manually using NIH (National Institutes of Health) ImageJ software (Bethesda, MD, USA) by an investigator blinded to treatment regimen. After counting, percent cell survival was obtained by plotting the total numbers of cells on the lesioned side as a percentage of those on the non-lesioned side of the same animal.

### Striatal biochemistry

Right hemispheres corresponding to the lesioned hemisphere were separated and homogenized in 1 ml of EGTA–glutathione (236.6 mM EGTA, 195.2 mM l-glutathione reduced, pH 7.0–7.4) in 1.5-ml microcentrifuge tubes. Samples were then spun in a tabletop centrifuge for 10 min at 12,000 r.p.m. Hundred μl of supernatant was drawn off and placed into a fresh, labeled 1.5-ml microcentrifuge tube and immediately placed at −20 °C. The remaining samples were placed at −20 °C. Hundred-μl aliquots were sent to Vanderbilt (Vanderbilt School of Medicine Neurochemistry Core) for high-performance liquid chromatography determination of tissue content of dopamine and its metabolites.

## Results

### P7C3 compounds protect rats from motor deficits after 6-OHDA exposure

**Figure 1:**
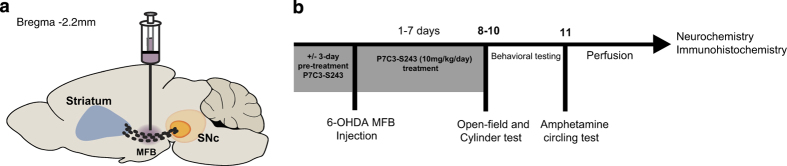
Experimental design. (**a**) Graphical illustration of the site of stereotaxic injection in the MFB shows dopaminergic fibers connecting the striatum and the SNc. (**b**) Schematic of the experimental design illustrates that rats received a unilateral stereotactic injection of 6-OHDA or saline in the MFB, followed by daily injection of P7C3-S243 for 7 days. Open field and cylinder tests were performed 8–10 days after 6-OHDA injection, and the amphetamine-circling test was conducted on day 11. Rats were transcardially perfused on day 14, and brain tissue was processed for neurochemistry and immunohistochemistry analysis. MFB, median forebrain bundle; 6-OHDA, 6-hydroxydopamine; SNc, substantia nigra pars compacta.

We first tested protective efficacy of P7C3 compounds in the 6-OHDA *in vivo* rat model of PD with 3 days of pretreatment with P7C3 compounds, as others have routinely applied this testing paradigm.^23^ We selected a test dose of 10 mg/kg/day intraperitoneally for P7C3 compounds, as we have previously shown that this concentration is effective in blocking MPTP toxicity^14,15^ and neurotoxicity after traumatic brain injury.^11,12^ P7C3-A20 or P7C3-S243 were administered daily for 3 days, followed by unilateral injection of 6-OHDA in the right MFB of adult male Long Evans rats (Figure 1a). Daily administration of P7C3 compounds was continued for the next 7 days, such that animals were treated both before and after toxin exposure, and behavioral testing was conducted 8–11 days after unilateral 6-OHDA lesion. Specifically, rats were evaluated for motor ability via three behavioral tests: assessment of rearing ability in the cylinder test, assessment of distance traveled in the open field test, and amphetamine-induced circling. In a separate experiment, the same testing paradigm was employed after unilateral 6-OHDA injection, but daily administration of P7C3-S243 was not initiated until 5–10 min after 6-OHDA exposure, while the rat was still under anesthesia, such that animals were treated only after toxin exposure (Figure 1b). The goal of this latter test was to evaluate efficacy of our putative protective compound in a preclinical model more relevant to the human condition, in which treatment would not be initiated until the disease process was already underway. Notably, no significant differences between these two different treatment groups (pretreatment only versus both pre- and post-treatment) in behavioral outcome measures were noted, reflecting a wide window of opportunity for therapeutic intervention with P7C3 compounds in this model of PD.

**Figure 2:**
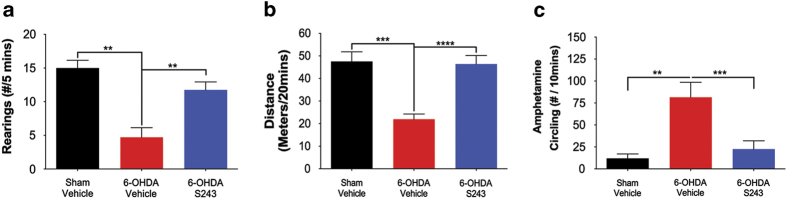
Post-treatment with P7C3-S243 protects rats from 6-OHDA-induced motor deficits. (**a**) Cylinder test showed a significant decrease in the total amount of rearings of 6-OHDA-exposed animals that received vehicle compared with sham-injured animals that received vehicle (**P<0.005; one-way ANOVA), and also to 6-OHDA-exposed animals that were treated with P7C3-S243 (***P<0.001; one-way ANOVA). (*n*=5 sham—vehicle, *n*=7 6-OHDA—vehicle, *n*=16 6-OHDA—P7C3-S243.) (**b**) Open field test showed a significant decrease in the total distance traveled of 6-OHDA-exposed animals that received vehicle compared with sham-injured animals that received vehicle (***P<0.001; one-way ANOVA), and also with 6-OHDA-exposed animals that were treated with P7C3-S243 (****P<0.0001; one-way ANOVA). (*n*=7 sham—vehicle, *n*=17 6-OHDA—vehicle, *n*=17 6-OHDA—P7C3-S243.) (**c**) Amphetamine-circling test showed a significant increase in ipsilateral (toward the lesion site) rotations of 6-OHDA-exposed animals that received vehicle compared with sham-injured animals that received vehicle (**P<0.005; one-way ANOVA), and also with 6-OHDA-exposed animals that were treated with P7C3-S243 (***P<0.001; one-way ANOVA). (*n*=5 sham—vehicle, *n*=7 6-OHDA—vehicle, *n*=16 6-OHDA—P7C3-S243.) ANOVA, analysis of variance; 6-OHDA, 6-hydroxydopamine.

The cylinder test allows measurement of rearings, which are scored as extension of hindlimbs beyond the typical grooming position while the rat places its paws on the walls of the cylinder in an exploratory manner.^21^ In the pre- and post-treatment experiment, rats displayed significantly fewer rearings/5 min when tested 8–10 days after 6-OHDA exposure, and treatment with P7C3-A20 alone, without toxin exposure, did not change the number of rearings relative to sham-injured animals. The number of rearings was restored to normal, however, when animals were both pre- and post-treated with either P7C3-A20 or P7C3-S243 (sham–veh, 15.0±1.140, *n*=5, **P=0.0072; sham-A20, 15.0±1.783, *n*=10, **P=0.0012; 6-OHDA–veh, 4.174±1.426, *n*=7; 6-OHDA–A20, 12.60±1.784, *n*=10, *P=0.0162; 6-OHDA–S243, 12.33±2.021, *n*=9, *P=0.0252; all *P* values were determined with one-way analysis of variance comparing with the 6-OHDA–veh group). Treatment of 6-OHDA-exposed rats with P7C3-S243 only after toxin exposure (post-treatment only) also restored the number of rearings to a normal level in 6-OHDA-lesioned animals (Figure 2a).

Similar to the cylinder test of rearing motor behavior, we noted that 6-OHDA-lesioned rats spontaneously traveled notably less distance in the open field, relative to sham-injured animals. In the pre- and post-treatment experiment, rats traveled significantly less in the 20-min test period in the open field chamber 8–10 days after 6-OHDA exposure, and treatment with P7C3-A20 alone, without toxin exposure, did not change the distance traveled. Locomotor activity was restored to normal, however, when animals were both pre- and post-treated with either P7C3-A20 or P7C3-S243 (sham–veh, 47.54±4.256, *n*=7, ***P=0.0003; sham-A20, 48.75±3.417, *n*=14, ****P<0.0001; 6-OHDA–veh, 21.95±2.326, *n*=17; 6-OHDA–A20, 38.17±5.633, *n*=9, *P=0.0198; 6-OHDA–S243, 34.98±4.595, *n*=13, *P=0.0441; all *P* values were determined with one-way analysis of variance comparing with the 6-OHDA–veh group). In addition, 6-OHDA-lesioned animals that were treated with P7C3-S243 only after toxin exposure (post-treatment only) also exhibited completely restored spontaneous open field motor activity (Figure 2b).

As a final test of motor behavior, we evaluated the same groups of rats in the amphetamine-induced circling test 11 days after 6-OHDA lesioning. Systemically administered amphetamine triggers catecholamine release while simultaneously decreasing catecholamine uptake and degradation, leading to augmentation of dopaminergic signaling in the brain that increases spontaneous motor behavior. After 6-OHDA-mediated unilateral lesioning of the SNc-striatal dopaminergic signaling system, amphetamine-driven motor activity results in ipsiversive circling directed toward the side of the lesion. In the pre- and post-treatment experiment, rats displayed 7–8 times more ipsiversive circling events than did sham-injured rats, or sham-injured rats treated with P7C3-A20. Amphetamine-driven ipsiversive circling was prevented, however, when animals were both pre- and post-treated with either P7C3-A20 or P7C3-S243 (sham–veh, 12.0±5.089, *n*=5, ***P=0.0009; sham-A20, 10.10±3.321, *n*=14, ****P<0.0001; 6-OHDA–veh, 81.57±16.93, *n*=7; 6-OHDA–A20, 33.0±10.26 *n*=9, **P=0.0084; 6-OHDA–S243, 28.40±10.17, *n*=10, **P=0.0028; all *P* values were determined with one-way analysis of variance comparing with the 6-OHDA–veh group). In addition, 6-OHDA-lesioned animals that were treated with P7C3-S243 only after toxin exposure (post-treatment only) showed levels of this parameter restored to those of normal sham-injured rats (Figure 2c). Typical examples of this protective effect on amphetamine-induced circling behavior are shown in the online video clips, consisting of three representative samples (Supplementary Video 1) of rats that received saline (the vehicle for 6-OHDA) and VEH (vehicle for P7C3-S243), three representative samples (Supplementary Video 4) of rats that received 6-OHDA and VEH (vehicle for P7C3-S243), and three representative samples (Supplementary Video 7) of rats that received 6-OHDA and P7C3-S243.

### P7C3 compounds protect rats from dopaminergic cell death after 6-OHDA exposure, and also preserve brain levels of dopamine and dopamine metabolites

**Figure 3:**
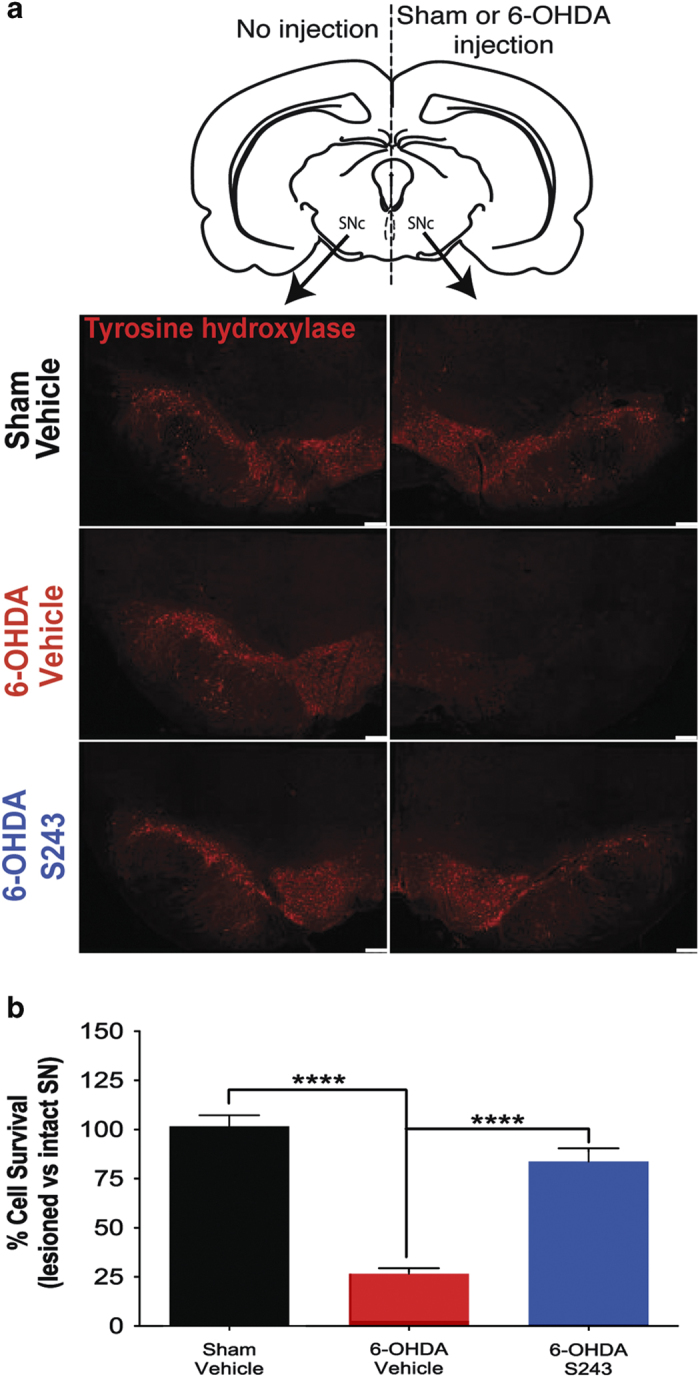
Treatment with P7C3-S243 protects rats from 6-OHDA-induced killing of dopaminergic neurons. (**a**) Representative immunohistochemistry of TH in the SNc shows that the non-injected side (left) shows no reduction in TH-positive cells in any group, whereas the injected side (right) shows prominent reduction in TH staining only in rats that were exposed to 6-OHDA and then administered vehicle. Scale bars=250 μm. (**b**) Quantification of TH-positive cells shows a significant decrease in rats that were exposed to 6-OHDA and then administered vehicle compared with sham-injured rats that received vehicle (****P<0.0001; one-way ANOVA), and also a significant increase in rats that were exposed to 6-OHDA and then treated with P7C3-S243 compared with the 6-OHDA–vehicle group (****P<0.0001; one-way ANOVA). (*n*=8 sham—vehicle, *n*=11 6-OHDA—vehicle, *n*=14 6-OHDA—P7C3-S243.) ANOVA, analysis of variance; 6-OHDA, 6-hydroxydopamine; SNc, substantia nigra pars compacta; TH, tyrosine hydroxylase.

To determine whether protection of motor behavior after 6-OHDA exposure by P7C3 compounds correlated with protection of SNc dopaminergic neurons from cell death, we perfused animals after behavioral testing to recover their brains. Brains were stained with antibodies specific to TH, the enzyme that catalyzes the conversion of the amino acid l-tyrosine to l-3-4-dihydroxyphenylalanine, which serves as the precursor for dopamine. TH staining thus provides a means to immunohistochemically identify dopaminergic neurons. By counting the number of TH-positive neurons, we were able to quantify the magnitude of dopaminergic neuronal cell death in the SNc. After 6-OHDA exposure, rats displayed a 75% reduction in TH-positive neurons in the ipsilateral substantia nigra, compared with the contralateral non-injected side (Figure 3a,b). When either P7C3-A20 or P7C3-S243 was administered both before and after toxin exposure, the level of TH-positive neurons was increased, with greater magnitude of effect noted in the P7C3-S243-treated animals (sham–veh, 101.6±5.653, *n*=8, ****P<0.0001; sham-A20, 106.1±2.619, *n*=5, ****P<0.0001; 6-OHDA–veh, 26.77±3.156, *n*=10; 6-OHDA–A20, 45.47±8.105, *n*=9, *P=0.012; 6-OHDA–S243, 69.57±7.170, *n*=13, ****P<0.001; all *P* values were determined with one-way analysis of variance comparing with the 6-OHDA–veh group). Notably, treatment with P7C3-S243 only after toxin exposure also achieved a protective effect that closely approximated that seen in sham-injured rats (Figure 3b).

**Figure 4:**
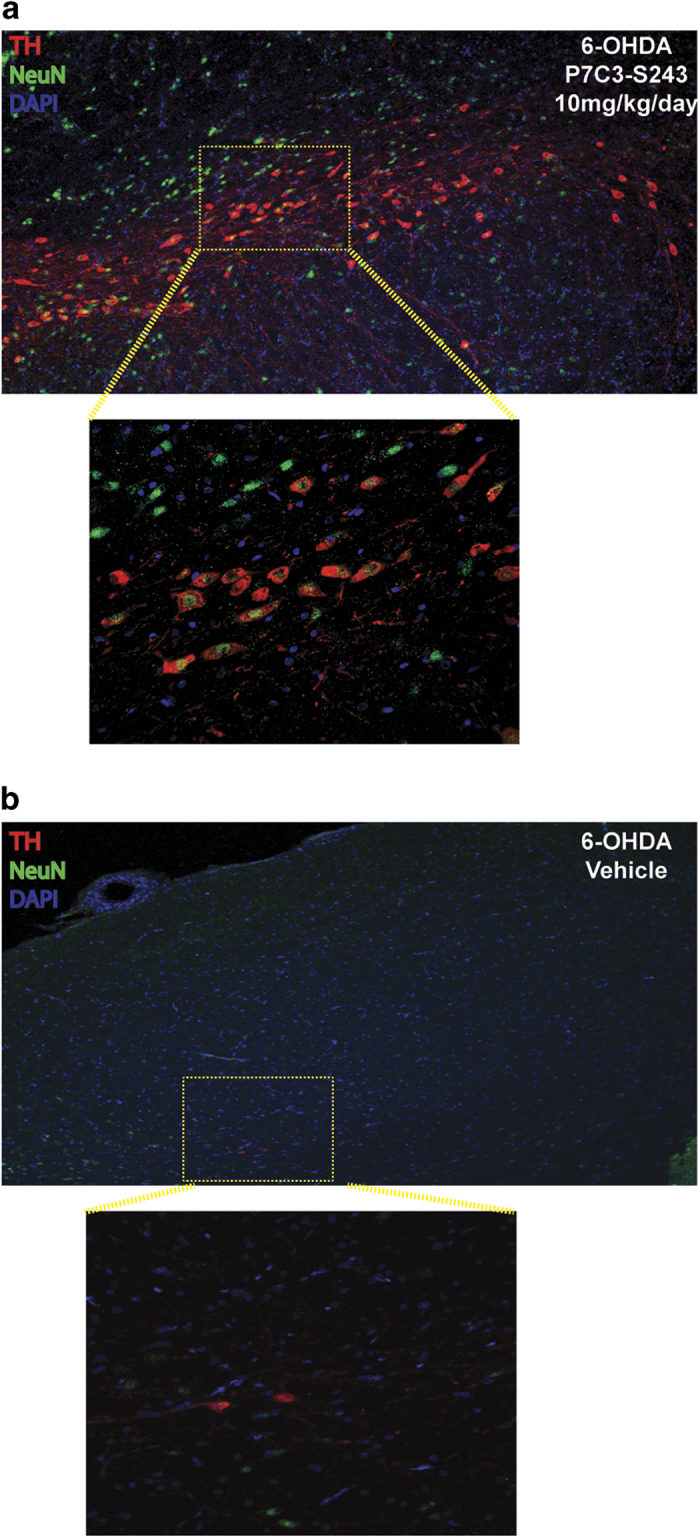
Treatment of 6-OHDA-exposed rats with P7C3-S243 preserves both TH and NeuN staining, illustrating that TH staining corresponds to neuronal survival. (**a**) Immunofluorescence of the SNc of a rat injected with 6-OHDA and then treated with P7C3-S243 (10 mg/kg/day) for 7 days shows preservation of colocalized TH and NeuN (top is low magnification and bottom is high magnification of the indicated region). (**b**) Immunofluorescence of the SNc of an animal injected with 6-OHDA and then administered vehicle for 7 days shows loss of both TH and NeuN labeling (top is low magnification and bottom is high magnification of the indicated region). DAPI was used as a nuclear marker. DAPI, 4ʹ,6-diamidino-2-phenylindole; 6-OHDA, 6-hydroxydopamine; SNc, substantia nigra pars compacta; TH, tyrosine hydroxylase.

To ensure that TH staining corresponded to neuronal survival, and not simply expression levels of TH in otherwise healthy neurons, we conducted co-immunostaining for TH and the neuronal-specific marker NeuN. As shown in Figure 4, immunofluorescence of the SNc of a rat injected with 6-OHDA and then treated with P7C3-S243 for 7 days showed preservation of colocalized TH and NeuN. By contrast, immunofluorescence of the SNc of a rat injected with 6-OHDA and then administered vehicle for 7 days showed loss of both TH and NeuN labeling. These results indicate that expression of TH corresponds with survival of SNc neurons.

**Figure 5:**
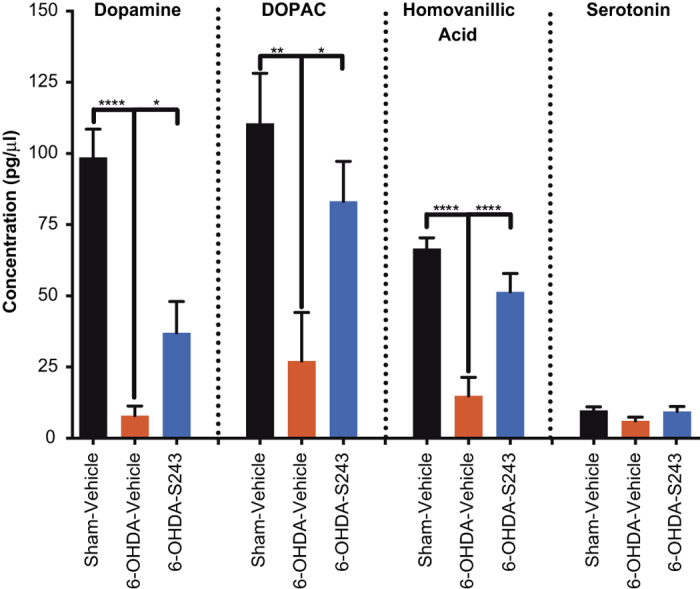
Treatment with P7C3-S243 protects rats from 6-OHDA-induced depletion of striatal dopamine and dopamine metabolites. HPLC analysis of striatal concentration of dopamine, DOPAC, and homovanillic acid showed a significant decrease in rats that were exposed to 6-OHDA and then administered vehicle compared with sham-injured rats that received vehicle, and also a significant increase in rats that were exposed to 6-OHDA and then treated with P7C3-S243 compared with the 6-OHDA–vehicle group. No difference was found in the concentration of serotonin in all groups (one-way ANOVA). (*n*=7 sham—vehicle, *n*=17 6-OHDA—vehicle, *n*=17 6-OHDA—P7C3-S243.) ANOVA, analysis of variance; HPLC, high-performance liquid chromatography; 6-OHDA, 6-hydroxydopamine.

As another measure of protective efficacy of dopaminergic signaling, we evaluated catecholamines using high-performance liquid chromatography from the ipsilateral striatum and forebrain of rats. After 6-OHDA lesioning, animals displayed a massive loss of dopamine and its metabolites DOPAC and homovanillic acid, with levels significantly restored toward normal in rats that were also post-treated with P7C3-S243 (Figure 5). As a control, we also measured levels of serotonin in all groups, and no significant differences were noted (Figure 5).

## Discussion

We have previously reported the discovery and development of the P7C3 class of neuroprotective molecules, which activate the rate-limiting enzyme in nicotinamide adenine dinucleotide salvage (nicotinamide phosphoribosyltransferase) and offer potent protective efficacy in a broad spectrum of preclinical models of neurodegenerative disease.^2,4,5^ One of the most prevalent and devastating forms of neurodegenerative disease affecting patients today is PD, and there is a significant unmet need for development of a medication that could slow or prevent PD progression by blocking death of dopaminergic neurons, which drives the disease process. Although we have previously demonstrated efficacy of P7C3 compounds in the mouse MPTP model of PD,^14,15^ in rodents this preclinical model suffers from a lack of robust behavioral motor deficits that represent the cardinal features of PD in humans. It is also essential to establish efficacy in multiple preclinical models of any human disease before initiating clinical trials in patients.

Accordingly, we have now extended our work to evaluate the efficacy of P7C3 compounds in the 6-OHDA rat model of PD, which is characterized by prominent motor deficits associated with unilateral loss of SNc dopaminergic neurons. We show here in rigorously blinded studies with large numbers of animals that P7C3 compounds protect rats from acquisition of side-biased motor deficits after exposure to 6-OHDA, in rearing, locomotion, and amphetamine-induced circling tests of motor activity. This protection is associated with the ability of P7C3 compounds to block dopaminergic neuron cell death in the SNc after 6-OHDA exposure, and this also correlates with preservation of brain levels of dopamine and dopamine metabolites. Efficacy was shown with administration of either P7C3-A20 or P7C3-S243 before and after 6-OHDA exposure, and also with administration of P7C3-S243 restricted to the post-6-OHDA exposure interval. This latter finding is particularly promising, in that patients are not treated until the disease has already begun. P7C3-S243 is also a promising compound for drug development, in that it is potently efficacious, lacks the aniline ring present in other active P7C3 derivatives, readily crosses the BBB, and can be prepared as a single active enantiomer.^11,12,15^ In summary, we propose that a properly optimized variant of the P7C3 class of neuroprotective chemicals, such as P7C3-S243, may offer promise for developing a novel treatment approach for treating patients suffering from PD.

## Notes

Supplementary Videos Information
