# Supplementary material for: Protective efficacy of P7C3-S243 in the 6-hydroxydopamine model of Parkinson's disease
Source: NPJ Parkinsons Dis. 2015 May 21;1:15010–. doi: 10.1038/npjparkd.2015.10 (PMC4859442; doi:10.1038/npjparkd.2015.10)
Supplement: Supplementary Videos Information [file npjparkd201510-s1.doc]

Supplementary Video 1 – Sham rat treated with vehicle shows no deficit in the methamphetamine–circling test

Supplementary Video 2 – Second sham rat treated with vehicle shows no deficit in the methamphetamine–circling test

Supplementary Video 3 – Third sham rat treated with vehicle shows no deficit in the methamphetamine–circling test

Supplementary Video 4 – 6-OHDA-exposed rat treated with vehicle shows multiple ipsiversive rotations in the methamphetamine–circling test

Supplementary Video 5 – Second 6-OHDA-exposed rat treated with vehicle shows multiple ipsiversive rotations in the methamphetamine–circling test

Supplementary Video 6 – Third 6-OHDA-exposed rat treated with vehicle shows multiple ipsiversive rotations in the methamphetamine–circling test

Supplementary Video 7 – 6-OHDA-exposed rat post-treated with P7C3-S243 (10mg/kg/day) shows protection in the form of fewer ipsiversive rotations than rats from the 6-OHDA-vehicle group in the methamphetamine–circling test

Supplementary Video 8 – Second 6-OHDA-exposed rat post-treated with P7C3-S243 (10mg/kg/day) shows protection in the form of fewer ipsiversive rotations than rats from the 6-OHDA-vehicle group in the methamphetamine–circling test

Supplementary Video 9 – Third 6-OHDA-exposed rat post-treated with P7C3-S243 (10mg/kg/day) shows protection in the form of fewer ipsiversive rotations than rats from the 6-OHDA-vehicle group in the methamphetamine–circling test
